# Native T1 Mapping Magnetic Resonance Imaging as a Quantitative Biomarker for Characterization of the Extracellular Matrix in a Rabbit Hepatic Cancer Model

**DOI:** 10.3390/biomedicines8100412

**Published:** 2020-10-13

**Authors:** Sarah Keller, Tabea Borde, Julia Brangsch, Lisa C. Adams, Avan Kader, Carolin Reimann, Pimrapat Gebert, Bernd Hamm, Marcus Makowski

**Affiliations:** 1 Department of Radiology, Charité—Universitätsmedizin Berlin, Corporate Member of Freie Universität Berlin, Humboldt-Universität zu Berlin, and Berlin Institute of Health, Charitéplatz 1, 10117 Berlin, Germany; tb.borde@gmail.com (T.B.); julia.brangsch@charite.de (J.B.); Lisa.adams@charite.de (L.C.A.); avan.kader@charite.de (A.K.); carolin.reimann@charite.de (C.R.); bernd.hamm@charite.de (B.H.); marcus.makowski@tum.de (M.M.); 2 Department of Diagnostic and Interventional Radiology, School of Medicine & Klinikum Rechts der Isar, Technical University of Munich, Munich (TUM), Ismaninger Str. 22, 81675 München, Germany; 3 Department of Biometry and Epidemiology, Charité—Universitätsmedizin Berlin, Corporate Member of Freie Universität Berlin, Humboldt-Universität zu Berlin, and Berlin Institute of Health, Charitéplatz 1, 10117 Berlin, Germany; pimrapat.gebert@charite.de

**Keywords:** magnetic resonance imaging, extracellular matrix, liver cancer, rabbits, VX2, T1 mapping

## Abstract

To characterize the tumor extracellular matrix (ECM) using native T1 mapping magnetic resonance imaging (MRI) in an experimental hepatic cancer model, a total of 27 female New Zealand white rabbits with hepatic VX2 tumors were examined by MRI at different time points following tumor implantation (day 14, 21, 28). A steady-state precession readout single-shot MOLLI sequence was acquired in a 3 T MRI scanner in prone position using a head-neck coil. The tumors were segmented into a central, marginal, and peritumoral region in anatomical images and color-coded T1 maps. In histopathological sections, stained with H&E and Picrosirius red, the regions corresponded to central tumor necrosis and accumulation of viable cells with fibrosis in the tumor periphery. Another region of interest (ROI) was placed in healthy liver tissue. T1 times were correlated with quantitative data of collagen area staining. A two-way repeated-measures ANOVA was used to compare cohorts and tumor regions. Hepatic tumors were successfully induced in all rabbits. T1 mapping demonstrated significant differences between the different tumor regions (*F*(1.43,34.26) = 106.93, *p* < 0.001) without interaction effects between time points and regions (*F*(2.86,34.26) = 0.74, *p* = 0.53). In vivo T1 times significantly correlated with ex vivo collagen stains (area %), (center: r = 0.78, *p* < 0.001; margin: r = 0.84, *p* < 0.001; peritumoral: r = 0.73, *p* < 0.001). Post hoc tests using Sidak’s correction revealed significant differences in T1 times between all three regions (*p* < 0.001). Native T1 mapping is feasible and allows the differentiation of tumor regions based on ECM composition in a longitudinal tumor study in an experimental small animal model, making it a potential quantitative biomarker of ECM remodeling and a promising technique for future treatment studies.

## 1. Introduction

The extracellular matrix (ECM) is a major component of tumoral stroma and has attracted much attention as a key regulator of cell and tissue function [1,2]. For a long time regarded as a mere mechanical link between cells, the ECM has since been shown to influence tissue migration, adhesiolysis, and repair through the regulation of biochemical and biophysical pathways [3,4]. While the production and degradation of proteins as a major component of the ECM are strictly balanced in healthy tissue, these processes often become dysregulated in cancer [5,6]. More specifically, the upregulation of collagen as the most abundant ECM component appears to play a prominent role in the tumor activating orchestra. For primary [7,8] and secondary [1,9,10] liver cancers, it has been shown that collagen upregulation seems to play a substantial role in angiogenesis, tumor cell infiltration and thus sensitivity to anticancer agents.

In recent years, native (unenhanced) T1 mapping has developed into a useful, noninvasive imaging method to quantitatively characterize tissue properties. T1 mapping measures longitudinal T1 relaxation time in milliseconds, which primarily depends on the molecular microenvironment of water molecules in the tissue. As a result, various changes in the extracellular space, such as increased water content in edema [11,12], protein deposition [13], or increased collagen formation [14,15], for example in fibrosis, can be detected using native T1 mapping.

Therefore, remodeling of the ECM with increasing collagen accumulation can be quantified by T1 mapping. T1 mapping is most commonly performed using the modified look-locker inversion recovery sequence (MOLLI). In the myocardium, MOLLI-based T1 mapping has shown excellent diagnostic performance with regard to the detection of tissue changes in patients compared to controls. The strongest correlations were found between the histopathologically quantified collagen volume fraction and T2 mapping results as an indicator of edema [16]. Based on these capabilities, T1 mapping has been increasingly used for the diagnosis and differentiation of a variety of cardiac diseases such as myocardial fibrosis, myocardial infarction, or myocarditis [14,17,18], as well as systemic disorders such as amyloidosis and siderosis [13].

In the liver, native T1 mapping has been successfully applied as an imaging biomarker for the diagnosis and grading of fibrosis, and thus as a predictor of clinical outcome [19,20,21]. In experimentally induced liver fibrosis in a rabbit model [22], T1 mapping showed promising results in differentiating early and advanced liver fibrosis histopathologically classified by METAVIR [23]. However, despite its clinical practicability and its capability to reliably differentiate tissue alterations, only limited data are available on the potential of native T1 mapping for the detection and characterization of tumors. In a recent preclinical study, Zormpas-Petridis et al. found T1 mapping to reliably distinguish regions with different levels of tumor cell differentiation in an experimental mouse model of neuroblastoma [24]. In patients, native T1 mapping was successfully used to identify higher-grade renal cell carcinoma histologically validated by the collagen volume fraction [25]. However, to the best of our knowledge, there is as yet no preclinical or clinical study that has systematically investigated native T1 mapping sequences to characterize liver tumors in terms of ECM composition.

Therefore, the objective of this study was to assess native T1 mapping in a VX2 hepatic tumor rabbit model at different time points after tumor implantation. It was hypothesized (1) that due to its signaling characteristics, native T1 relaxation times are higher in the necrotic tumor center compared to the tumor margin and peritumoral tissue; (2) that native T1 mapping as a noninvasive, contrast-free tool reliably distinguishes tumor areas from normal liver tissue; and (3) that native T1 times correlate with collagen content in histopathological sections.

## 2. Experimental Section

### 2.1. Animal Model

This study was approved by the responsible authority and was conducted in accordance with local guidelines and provisions for the implementation of the Animal Welfare Act and regulations of the Federation of Laboratory Animal Science Associations (FELASA; registration number 0178/17; date 06/11/2017) and designed in accordance with the ARRIVE guidelines. The unenhanced magnetic resonance imaging (MRI) data used here were acquired as part of a larger study evaluating different contrast agents in the rabbit model. Twenty-seven female New Zealand white rabbits (Charles River Laboratories, Sulzfeld, Germany) aged 11 to 17 weeks with a mean weight (standard deviation) of 3.3 (0.3) kilograms were used for this experimental study. All animals were maintained in laminar flow rooms at constant temperature and humidity, with food and water provided ad libitum. VX2 cells were injected in the hindlimb of eight female donor rabbits and grown for 21–30 days as previously described [26]. The tumors were harvested and processed; approximately 4 mL of the resulting tumor chunks were subsequently implanted into the left liver lobe of the recipient animals by mini laparotomy. Donor rabbits received a perioperative anesthesia of medetomidine hydrochloride (cepetor, 0.25 mg/kg), and ketamine hydrochloride (ketamin, 30 mg/kg) subcutaneously. Carprofen (rimadyl, 4.0 mg/kg) was injected as an analgesic for three days following surgery.

**Figure 1:**
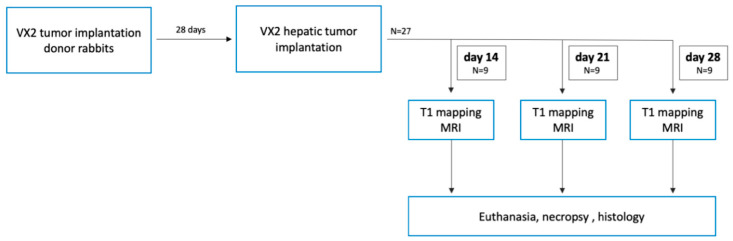
Experimental in vivo study design. In the vertical direction, the flow chart illustrates the VX2 rabbit tumor model and magnetic resonance imaging (MRI) on sequential time points. Groups of N = 9 tumor-bearing rabbits were assigned to one time point (day 14, 21, and 28 after implantation) and euthanized following the scan.

According to the study protocol (Figure 1), the rabbits were divided into three subgroups with N = 9 animals that were examined by MRI with T1 mapping on day 14, day 21, or day 28 after intrahepatic tumor implantation. Immediately after MRI, the rabbits were euthanized with pentobarbital sodium (Narcoren, 300 mg/kg body weight) under general anesthesia and necropsied. Tumor and liver parenchyma samples were collected and processed for histopathological analysis.

### 2.2. MR Imaging

MRI was performed in deep sedation in a 3 T clinical scanner (mMR Biograph, Siemens Medical Solutions, Erlangen, Germany). The rabbits were imaged in prone position with a clinically approved head-neck coil. Anatomic images were acquired using a T2-weighted (TR/TE 5500/90 ms, voxel size 0.5 × 0.5 × 3.0 mm^3^, FOV 180 × 180 mm^2^) and T1-weighted Dixon sequence (TR/TE 4.76/1.49 ms, voxel size 0.5 × 0.5 × 2.0 mm^3^, FOV 272 × 272 mm^2^) followed by a commercially available steady-state precession readout single-shot MOLLI sequence (TR/TE 1155/2.45 ms, voxel size 1.0 × 1.0 × 3.0 mm^3^, FOV 250 × 250 mm^2^). The total scanning time was approximately 12 min. T1 maps were automatically computed on a pixel-by-pixel basis. The resulting pixel-by-pixel maps were displayed by use of a customized 12-bit lookup table, and the color map was visible immediately after data acquisition. In the color map, the signal intensity (SI) of each pixel reflects the absolute T1 value of the underlying value.

### 2.3. Image Postprocessing

Imaging datasets were evaluated with the open source software tool Horos (version 4.0.0.0RC1, Nimble Co LLC, Annapolis, MD, USA). Regions were manually segmented using the anatomical T1-weighted sequences in conjunction with T2-weighted images into a central, marginal, and peritumoral region.

**Figure 2:**
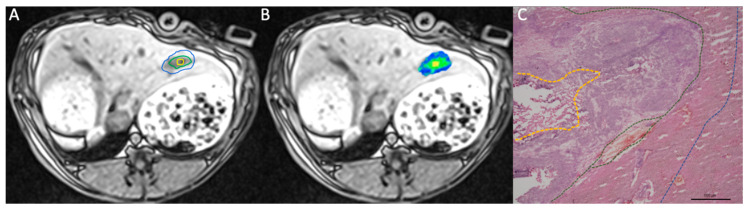
Schematic illustration of manually segmented tumor regions. (**A**) Axial T1-weighted images were used for delineation of the tumor center (yellow), margin (green), and the peritumoral region (blue). (**B**) Corresponding native MOLLI color-coded map depicting regional T1 times. (**C**) Corresponding segmentation in the histopathological H&E-stained sections.

According to preliminary studies [27,28], central necrosis has low T1 and low T2 signal intensity. Viable tumor tissue is characterized by slight T2 hyperintensity and can therefore be discriminated well from necrosis and surrounding peritumoral tissue [28] (Figure 2). The peritumoral region was defined as an area around the tumor of approximately 2 mm. The respective ROI was then copied to the axial color-coded T1 map, using an automatic co-registration tool and by visual correlation in the case of breathing artifacts (Figure 2). In the respective histological hematoxylin and eosin (H&E) stains, the central region covered the tumor necrosis, while the marginal area included viable tumor cells and the fibrous tumor capsule. The peritumoral region covered the peritumoral matrix including hepatocytes, inflammatory cells, stromal cells, etc. ROIs placed in healthy liver tissue and the autochthonous back muscles of the same image plane served as controls.

### 2.4. Histology

Following MRI, liver tumors were harvested for necropsy. Liver tumors were explanted and immediately frozen at −80 °C. Tissues were embedded and stored using embedding medium for cryostat sectioning (Tissue-Tek, Sakura Finetek, Torrance, CA, USA), cut at −20 °C into 10 µm sections and mounted on adhesion slides (SuperFrost Plus, Thermo Scientific, Waltham, MA, USA). The sections were then stained with Picrosirius red stain and H&E to visualize collagen content and tumor composition. A light microscope (BzX800, Keyence, Japan) was used for the examination of the slides. Representative magnification (×2 and ×10) digitalized images (TIFF file format) were stored for computer-assisted image analysis using the open-access software ImageJ (ImageJ software, version 1.51, Wayne Rasband, National Institutes of Health; https://imagej.nih.gov/ij/, Bethesda, MD, USA). For image analysis of histologies, separate ROIs were drawn manually, as these deviated slightly due to the fixation process and therefore did not correlate with the MR images. The color profile of the corresponding region was automatically segmented. The percentage of collagen fibers per region was determined by dividing the percentage by the total area of the region.

### 2.5. Statistical Analysis

Since the end-points are semiquantitative in nature, a size of N = 9 was chosen as the balance between scientific necessity and resources available to perform the study in agreement with mandatory Animal Care and Use Committee limitations in USDA-restricted species, making it necessary to keep the numbers of animals used as low as reasonably achievable to meet study goals. All quantitative T1 mapping parameters (central, margin, peritumoral, liver) are expressed as mean and standard deviation (SD). Normal distribution was tested using normal QQ plots. Two-way repeated-measures ANOVA was performed to compare the change inT1 times between regions over time using Greenhouse–Geisser correction and Sidak’s post hoc tests in SPSS (v.25, IBM, Armonk, NY, USA). Univariate correlations were calculated using Pearson’s correlation, and the correlation coefficient (r) was presented. An α < 0.05 was considered statistically significant.

## 3. Results

**Figure 3:**
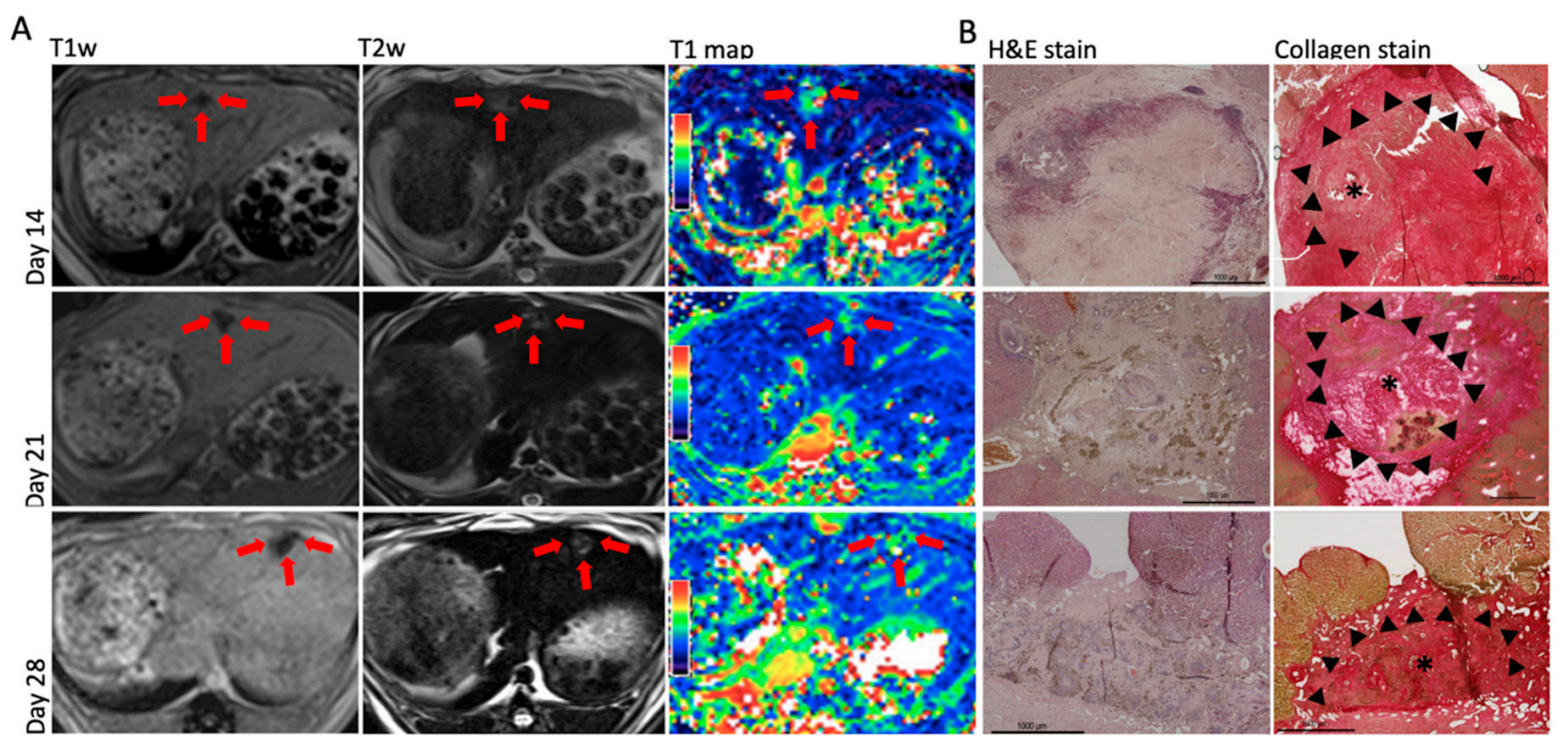
MRI and histological findings in experimentally implanted VX2 tumors at different time points (day 14, 21, and 28). (**A**) Axial MRI of implanted hepatic VX2 tumors (unenhanced T1wI, T2wI, color-coded T1map; (**B**) corresponding histopathological analysis: H&E stain and collagen stain. The asterisk marks the tumor center, the arrowheads the tumor margin.

Growth of single hepatic tumors was successfully induced in all rabbits. Mean tumor size was (mean (SD)) 11 (3) mm and increased slightly between day 14 (8 (2) mm) and day 28 (12 (2) mm). The intrahepatic tumors showed a hypointense signal in unenhanced T1 images and were slightly hyperintense in T2 images. The anatomical images correlated well with the color-coded T1 maps in which the tumors were demarcated by their high signal from surrounding liver parenchyma (Figure 3).

### 3.1. Native T1 Mapping

Consistent with the image impression in the color-coded T1 map, averaged across all time points, the highest native T1 relaxation times were found in the tumor center (SD) (1721.7 (488.6) ms), followed by the tumor margin (1198.7 (291.3) ms). Shorter T1 times were found in the peritumoral region (779.7 (212.1) ms). Overall, all tumor-associated T1 times were above healthy liver parenchyma (525.9 (185.5) ms).

**Figure 4:**
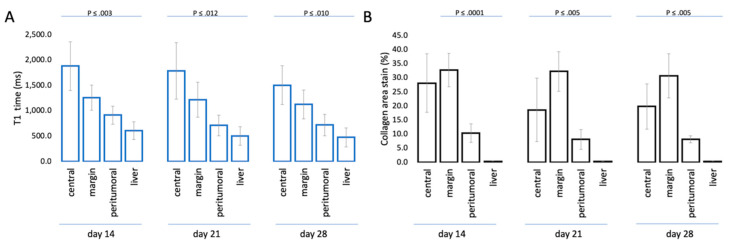
Association of T1 mapping and collagen staining in all animals. (**A**) Mean with standard deviation error bar of T1 times in different tumor areas 14, 21, and 28 days following tumor implantation; (**B**) mean with standard deviation error bar of the percentage collagen area stain (%) (Picrosirius red stain) at the three time points.

T1 mapping demonstrated significant differences between the segmented tumor center and margin as well as the peritumoral region and the liver parenchyma (*F*(1.43, 34.26) = 106.93, *p* < 0.001). There was no interaction effect between times and regions (*F*(2.86, 34.26) = 0.74, *p* = 0.53), nor an effect of time (*F*(2, 24) = 2.14, *p* = 0.14). T1 relaxation times were highest in the tumor center [mean (SD)] [1721.8 (488.6) ms] and decreased towards the periphery (margin: 1198.7 (291.3); peritumoral: 779.7 (212.1) ms; liver: 525.8 (185.5) ms) (Figure 4a). Post-hoc tests using Sidak’s correction revealed significant differences between all three tumor regions (*p* < 0.001) (Table 1, Figure 4). T1 relaxation times in the tumor center and margin decreased slightly but not significantly over time.

### 3.2. Histopathological Analysis

The tumor areas were visualized in collagen-stained sections and corresponded to the respective slight in the acquired MR images (Figure 3b). We consistently found significant (*p* < 0.001) differences in collagen content in the regions investigated at all time points. The highest relative proportions of collagen fibers were consistently found in the tumor margin (31.8 (6.5)%) followed by the tumor center (22.8 (9.8)%). The peritumoral region showed the lowest collagen content (9.2 (2.5)%); however, this was still significantly higher compared to healthy liver parenchyma (0.23 (0.18)%).

**Figure 5:**
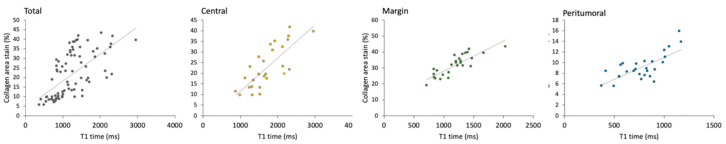
Association of T1 relaxation times and collagen area stain shown for all time points and areas.

The in vivo T1 relaxation times of all segmented regions together correlated with histopathological ex vivo collagen stain area (r = 0.64, *p* < 0.001). Specifically, the T1 times of the marginal regions showed the highest correlation coefficient (r = 0.84, *p* < 0.001), followed by the central (r = 0.78, *p* < 0.001), and peritumoral (r = 0.73, *p* < 0.001) regions (Figure 5).

## 4. Discussion

The main result of this study is that native T1 mapping based on specific T1 relaxation times allows the differentiation of tumor composition and the peritumoral region, which is not possible in such detail using conventional MR imaging. Native T1 mapping directly correlated with tumoral collagen content, making it a potential quantitative biomarker for ECM remodeling in hepatic tumors.

T1 longitudinal relaxation time is an intrinsic reflector of structural tissue composition in health and disease. The advent of the MOLLI pulse sequence has enabled fast and immediately available quantification of T1 tissue relaxation times in a single breath hold and thus facilitated clinical translation [29]. Currently, clinical T1 mapping is primarily used in cardiac imaging and has recently been incorporated into clinical cardiovascular guidelines. Further application areas have emerged with the use of T1 quantification to estimate liver function and the severity of liver fibrosis in humans [19,20,21] and animal models [22]. A recent study demonstrated superior diagnostic accuracy of T1 mapping over ultrasound elastography in early liver fibrosis [30].

Very few studies have so far investigated native T1 mapping to characterize the degree of tumor differentiation in high-grade renal cell carcinoma and neuroblastoma [24,25]. Data on native T1 mapping in clinical or experimental HCC are poor, and studies on the characterization of ECM composition using the MOLLI sequence are missing. In conjunction with a hepatocyte-specific contrast agent (Gd-EOB-DTPA), T1 mapping was shown to successfully differentiate various kinds of focal liver lesion based on morphologic imaging appearance. Using the percentage reduction T1 relaxation time (T1d%) in the hepatobiliary contrast agent phase combined with discriminant analysis, focal nodular hyperplasia, HCC, and cavernous hemangioma were distinguished with high sensitivity and specificity [31]. T1d% in the hepatocellular contrast agent phase also reliably predicted the degree of differentiation of HCC in the clinical setting [32].

Compared with published preliminary results of T1 mapping in the liver, quantitative T1 relaxation times measured without contrast agent administration show greater variation. For example, previous quantitative T1 mapping studies in the clinical setting calculated lower native T1 relaxation times (1008.6 (357.5) ms) for focal HCC compared to the values we obtained in a VX2 rabbit model. However, it must be noted that the study of Peng et al. [31] did not segment different tumor regions but averaged values across the whole tumor, which explains greater variability. Given the inherent tumor core necrosis of HCC, this approach might not be entirely representative in characterizing this type of tumor. Native T1 relaxation times found in a rabbit model of liver fibrosis by Li et al. [22] were very low (250.07 (88.12) ms) in the healthy liver parenchyma of control animals using nonenhanced liver acquisition volume acceleration (LAVA) with variable flip angle. In a recent study [30] quantifying fibrosis in a rat model using a look-locker sequence with an inversion recovery pulse comparable to our study, T1 relaxation times calculated for healthy liver parenchyma were similar to the values found in our study (525.8 (185.5) ms). The discrepancy with the aforementioned study may be attributable to technical sequence differences (e.g., LAVA versus MOLLI).

Native T1 values reflect the composite water signal from cells and extracellular space [33]. Higher native T1 values are primarily attributable to an increase in interstitial space, such as collagen, or the presence of edema [29]. In addition, other factors were shown to influence T1 relaxation such as inflammation, iron concentration, and steatosis, with higher T1 relaxation times in inflammation being primarily associated with interstitial edema [34,35,36]. In our study, we measured prolonged T1 times in the tumor center with decreasing values towards the peritumoral region and the shortest T1 relaxation times in the liver parenchyma. This distribution of T1 times corresponds to the histopathological findings of central necrosis and higher tumor cell counts and fibrosis in the tumor margin. Published results on the hepatic VX2 tumor model for comparison are not available. In the setting of invasive tumor proliferation, unrestraint angiogenesis and chronic inflammation may be the two major factors that affect T1 relaxation times. The discrimination between tumor center and tumor margin may be explained by inherent central tumor necrosis frequently already present in early HCC. The longer T1 times in the less collagen-rich tumor center can be explained by the fact that water accumulation in biological tissues results in greater increases in T1 times than scarring or fibrosis [29]. Similar to acute myocardial infarction, cellular destruction by necrosis leads to higher water content and surrounding interstitial edema and consecutively to a focal increase in T1 relaxation times [33]. The good demarcation of the tumor margin can be explained in part by tumor hypervascularity along with an increased collagen content. Tumor neoangiogenesis is especially pronounced in the viable tumor rim, lengthening longitudinal relaxation time and thus contributing to demasking the tumor in T1 maps [31]. The increase in water content due to central necrosis and concomitant inflammation may have an additive effect on relaxation times that exceeds the effect of the vascular and collagen network in the tumor margin and may also partly affect the tumor rim, thus contributing to good overall detection. An interesting and important result of our study is that T1 mapping also distinguishes the peritumoral region, which tends to be occult in conventional T1 and T2 images, from adjacent healthy liver parenchyma. This finding is of particular interest for interventional studies on the model, which investigate the inflammatory response in the peritumoral ECM as a possible sign of therapy response.

Collagen—the primary ECM protein—has been found to play a key role in the promotion of tumor angiogenesis and tumor cell proliferation [3,4]. Furthermore, previous investigators have described the stimulation of abnormal collagen synthesis with the expansion of the collagen matrix in tumors. The higher extracellular collagen content prolongs longitudinal relaxation times and is directly proportional to the increase in tumoral T1 relaxation time [37]. Nakamori et al. demonstrated the ability of native T1 mapping to reliably detect and quantify the histological collagen volume fraction in the heart [15]. In this study, there was a direct correlation between native T1 relaxation times and extracellular volume with the biopsy-proven collagen volume fraction. Consistent with these results, we found a direct correlation of native T1 relaxation times with the histologically proven collagen content in the three hepatic tumor regions investigated. These results also provide valuable information on the composition of the tumor-associated ECM. Since the latter has already been linked with tumor aggressiveness and growth, native T1 relaxation times may be used as a potential quantitative parameter for the evaluation of ECM remodeling in response to both local and systemic therapies.

Our study has some limitations. MRI was performed in free breathing but in deep sedation, which considerably reduces respiratory motion artifacts. Overall tumor size was relatively small and varied across animals. Greater variability in tumor size has been shown for this rabbit model before and seems to be related to both necrosis rate and external factors [38]. In our study, interindividual variability in tumor composition, regarding the collagen content and central necrosis, as well as the tumor growth, was evident. However, systematic characterization of tumor variability and development was beyond the scope of our study, which focused on the feasibility of the MOLLI sequence for tumor tissue characterization in the experimental model. Furthermore, histologic fixation can distort anatomy, which may thus deviate from anatomy on images acquired in vivo. ROIs were therefore, as already done in preliminary studies [27], manually reconstructed for analysis as direct correlation of thistological sections with MR images is not possible. Furthermore, scarring may have a similar effect as increased collagen accumulation [39]; however, the sequence used cannot differentiate scarring from ECM expansion by excessive collagen production. Invasive tumor chunk implantation increases the risk of scarring, which may have affected T1 relaxation times in our experiments. However, there was no histopathological evidence of excessive scarring. Finally, as already mentioned above, T1 relaxation times are influenced by other factors such as iron concentration and steatosis [34,35,36]. While liver steatosis is unlikely in the young rabbits used in our study, a more accurate quantification of T1 relaxation times would probably be possible by simultaneous measurement of liver and tumor iron concentrations. This effect could be investigated further in future studies of T2* and T1 mapping in the VX2 tumor model.

## 5. Conclusions

Native T1 mapping is a reliable noninvasive method to quantify ECM components in different areas of hepatic VX2 tumors and thus contributes to the identification and characterization of primary liver tumors. Therefore, it can be used as a quantitative biomarker of ECM remodeling and a promising technique for future treatment studies.

## Figures and Tables

**Table 1:** Discriminatory power of T1 times (ms) between the tumor (center, margin) and peritumoral regions averaged for all time points.

Time Point	Region		Mean Difference (R1–R2) (ms)	95% Confidence Interval (CI)	*p* Value *
	R1	R2			
**Day 14**	center	margin	620.1	314.1–926.1	<0.001
		peritumoral	965.6	486.9–1444.4	<0.001
		liver	1272.8	778.6–1767.1	<0.001
	margin	peritumoral	345.5	100.4–590.7	<0.003
		liver	652.7	337.0–968.1	<0.001
	peritumoral	liver	307.2	134.5–479.9	<0.001
**Day 21**	center	margin	569.1	263.1–875.1	<0.001
		peritumoral	1074.7	595.9–1553.4	<0.001
		liver	1284.0	789.7–1778.2	<0.001
	margin	peritumoral	505.6	260.4–750.7	<0.001
		liver	714.8	399.2–1030.5	<0.001
	peritumoral	liver	209.3	36.6–382.0	0.012
**Day 28**	center	margin	379.7	73.7–685.8	0.010
		peritumoral	785.5	306.7–1264.2	0.001
		liver	1030.7	536.4–1524.9	<0.001
	margin	peritumoral	405.8	160.7–650.9	<0.001
		liver	650.9	335.2–966.6	<0.001
	peritumoral	liver	245.1	72.5–417.8	0.003

* Adjusted *p*-value using Sidak’s method.
